# Small, Depressed-Type Early Colon Cancer Invading Shallow Submucosal Layer With Extensive Lymph Node Metastasis: A Case Report

**DOI:** 10.4021/gr304w

**Published:** 2011-05-20

**Authors:** Yurika Kawamura, Naotaka Ogasawara, Mari Mizuno, Makoto Sasaki, Yoshitsugi Ito, Yoshihiro Kondo, Hisatsugu Noda, Shinya Izawa, Masahiko Miyachi, Kunio Kasugai

**Affiliations:** a Department of Gastroenterology, Aichi Medical University School of Medicine, 21 Karimata, Yazako, Nagakute-cho, Aichi 480-1195, Japan; b Department of Surgery, Aichi Medical University School of Medicine, 21 Karimata, Yazako, Nagakute-cho, Aichi 480-1195, Japan

**Keywords:** Bevacizumab, Depressed type, Early colorectal cancer, Lymph node metastasis, mFOLFOX6

## Abstract

Early colorectal cancers are defined as invasive tumors that are limited to the mucosal layer or submucosal layer (SM), regardless of the presence or absence of lymph node (LN) metastasis. The reported incidence of LN metastasis of SM1 colon cancers is 0 - 5.9%, but the incidence in SM2 and SM3 colon cancers could be as high as 11.3 - 25.0%, and risk factors for LN metastasis include depth of SM invasion, growth patterns (polypoid or non-polypoid), histological sub-classification (moderate or poor differentiation) and regional lymphatic and vascular invasion. Among colorectal cancers with non-polypoid growth, the malignant potential is higher for depressed, than polypoid types, even for small tumors. Herein, we describe a patient with small, depressed-type early colon cancer with extensive LN metastasis and superficial SM invasion (pSM 450 µm). Six courses of chemotherapy with mFOLFOX6 and bevacizumab reduced the size of the LN metastases, thus eliciting a partial response (PR) according to the response evaluation criteria in solid tumors (RECIST).

## Introduction

Most early colon cancers are polypoid type, which undergo the adenoma–carcinoma sequence and have relatively slow growth rates. However, non-polypoid types of early colon cancer grow rapidly [1] and are classified as slightly elevated, lateral spreading tumors (LST) and depressed types [2]. Some non-polypoid cancers might arise *de novo* in the absence of an association with adenoma [3]. Flat or depressed types of early colon cancer have been identified in Japan, and the malignant potential of these types might be higher than that of early polypoid colon cancers [4]. Moreover, the incidence of submucosal layer (SM) invasion is higher in early colon cancers with depression (IIc, IIc+IIa, and IIa+IIc), than without depression or in the elevated type (Ip, Isp, and Is) [5]. One report indicates that the rates of SM invasion are 2.1%, 0.05%, 8.2% and 29.5% for polypoid, small flat adenoma, LST and depressed lesions, respectively [2]. Kudo et al. reported that vertical invasion of the SM between the muscularis mucosae and muscularis propria could be separated into three grades (SM1, SM2 and SM3: infiltration into the upper, middle and lower thirds of the SM, respectively) and this classification has been useful for treating early colon cancers [6]. A retrospective study of surgically removed SM colon cancers found that lymph node (LN) metastasis is significantly associated with the level of tumor invasion within the submucosa [7]. The rates of LN metastasis for tumors invading the upper (SM1), middle (SM2), and lower (SM3) thirds of the SM, are 2%, 9%, and 35%, respectively [7]. Cancers limited to the mucosal layer or SM1 can be safely and curatively treated by endoscopic mucosal resection (EMR) based on these results. Many investigators have demonstrated that LN metastasis is closely associated with depth of SM invasion, type of growth (polypoid or non-polypoid), lymphovascular invasion and histological sub-classification at the deepest invaded portion [3, 8-10]. Generally, poorly differentiated carcinomas have a high risk of LNs metastasis as well as distant metastasis [11].

This report described a 52-year-old man diagnosed with a small (pSM 450 µm) invasive, histologically mixed (moderately and poorly differentiated) lesion of depressed type early colon cancer with extensive LNs metastasis. Chemotherapy with mFOLFOX6 (Fluorouracil, 5-FU; Leucovorin, LV; oxaliplatin, L-OHP) plus bevacizumab elicited a partial response (PR) in extensive LN metastatic tumors.

## Case Report

**Figure 1:**
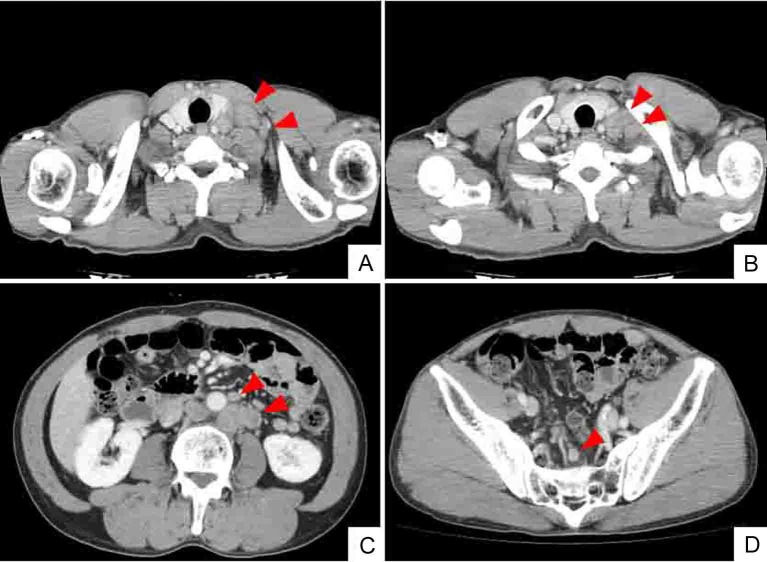
Whole body CT (computed tomography) shows mass of swollen left cervical (A) and supraclavicular (B) LNs. CT findings also indicate enlarged abdominal periaortic, inferior mesenteric and surrounding (C), and colic (D) LNs (arrowheads). However, CT findings do not indicate sigmoid colon tumor.

**Figure 2:**
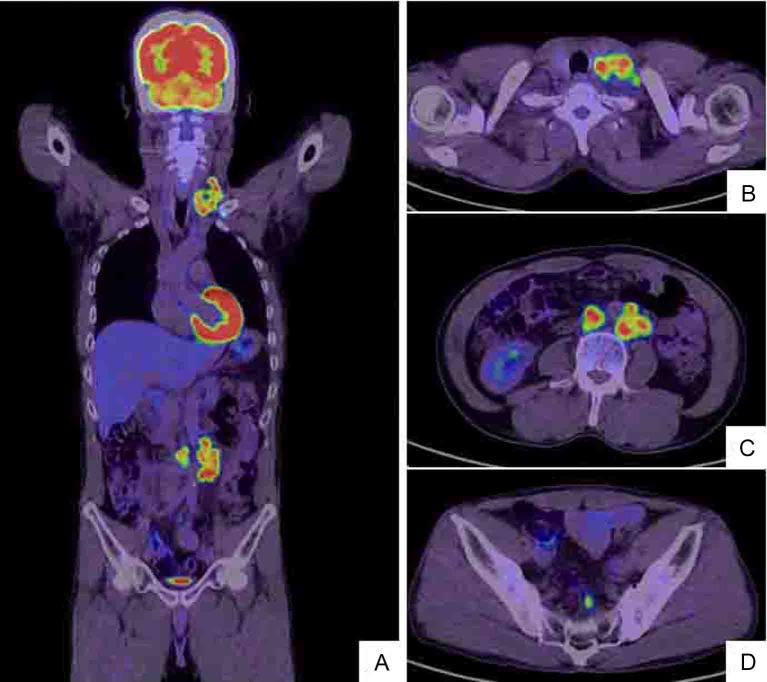
Whole body positron emission tomography (PET)/CT scan (A) shows hot lesions of multiple LN metastases to left cervix and left supraclavicular fossa (B) as well as enlarged left abdominal periaortic, inferior mesenteric and surrounding (C) and colic (D) LNs. The PET/CT and CT findings are identical (Fig. 1).

**Figure 3:**
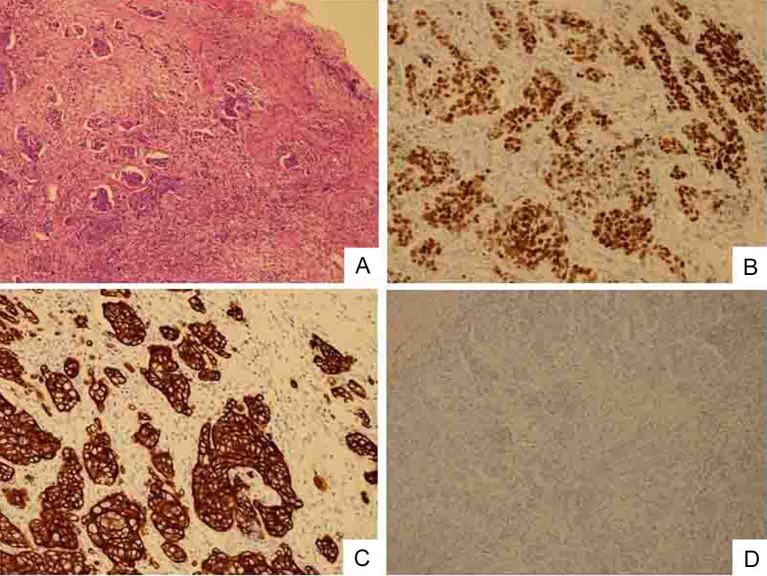
Biopsy specimen of swollen left clavicular LN. Cells are poorly differentiated adenocarcinoma. (A) HE staining (original magnification, × 100). Immunostaining shows that cancerous cells have nuclear expression of Cdx2 protein (B), intense and widespread positivity for CK20 protein (C) and negative immunostaining for CK7 (D).

**Figure 4:**
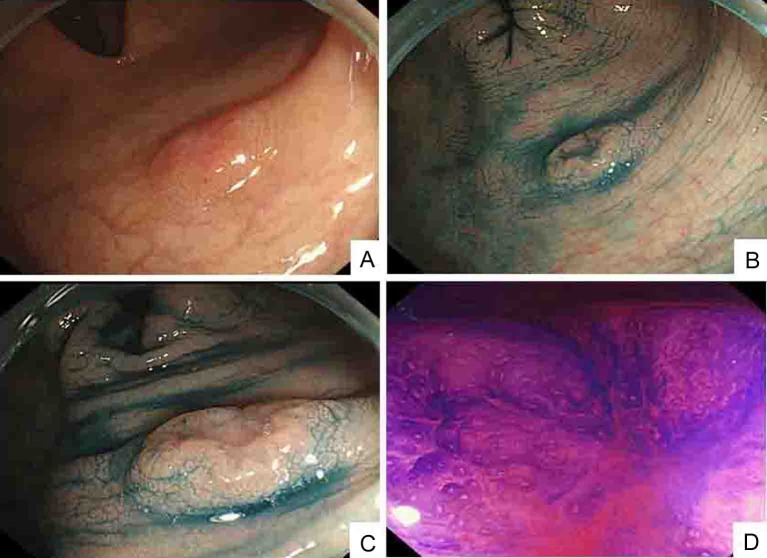
Colonoscopic findings: (A); indigo carmine contrast spray (B and C) demonstrate depressed type lesion 10 mm in diameter with distinct margins and marginal elevation (IIa+IIc type); crystal violet contrast spray (D) reveals pit pattern in central portion of tumor indicating V_I_ type.

**Figure 5:**
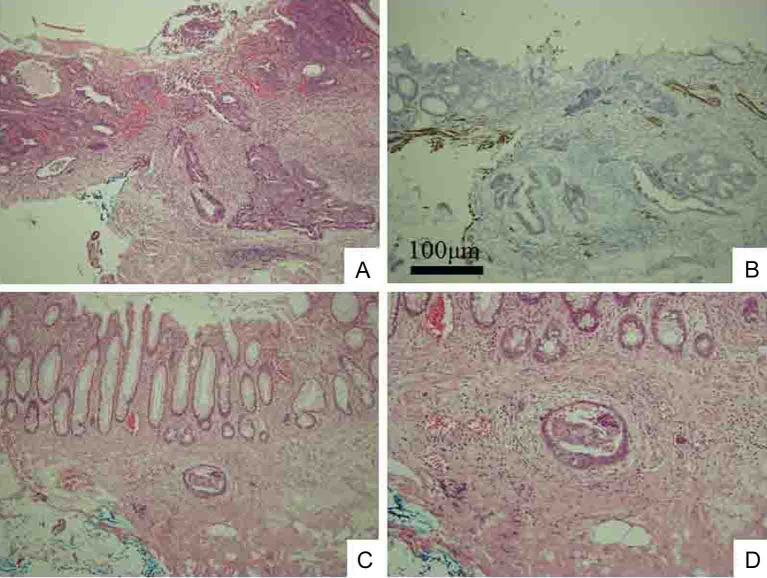
Histological examination shows moderately and poorly differentiated adenocarcinoma without adenoma component, indicating de novo development (A). Immunohistochemistry of desmin shows cancerous invasion of shallow SM to 450 µm from muscular layer of mucosa (B). Lymphovascular permeation of peritumoral submucosal lymphatics (C). Higher magnification of C (D). Original magnification: A, B, and C × 100; D × 200.

**Figure 6:**
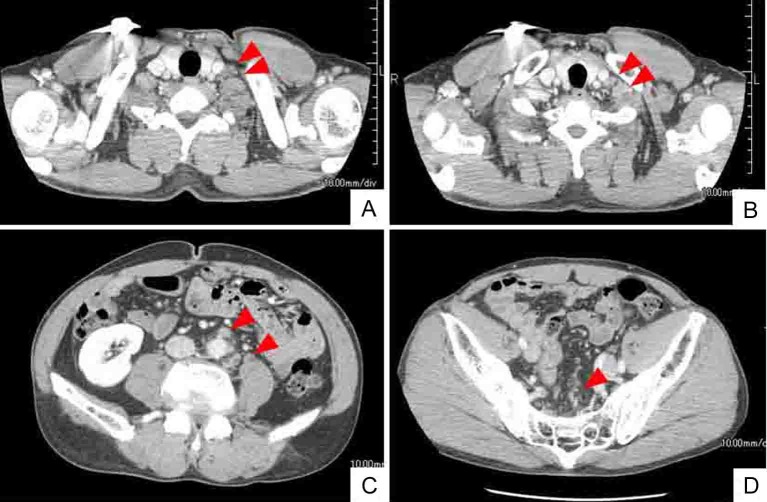
Whole body CT after 6 courses of chemotherapy (mFOLFOX6 and bevacizumab) shows smaller or disappeared metastatic LNs (arrow heads). Left cervical (A) and left supraclavicular (B) LNs. Abdominal periaortic, inferior mesenteric and surrounding (C), and regional colic (D) LNs.

**Figure 7:**
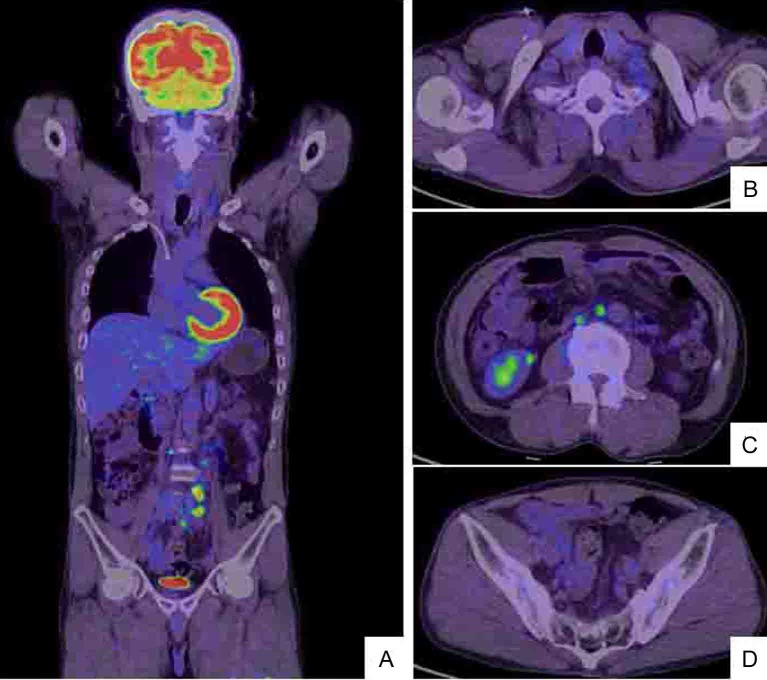
After 6 courses of the chemotherapy (the combination of mFOLFOX6 and bevacizumab), whole body PET/CT scan (A) demonstrates that the left cervical metastatic LNs (B) and the abdominal periaortic LNs are reduced in size and the intensity of hot lesions is obviously decreased (C). The regional colic LNs (D) are not recognized as hot lesions.

A 52-year-old man with no significant medical history presented with a tumor of the left cervix. A physical examination by computed tomography (CT) revealed that the tumor on the left side of the neck comprised a complex of swollen left cervical and clavicular LNs (Fig. 1). The CT findings simultaneously revealed swollen colic, abdominal periaortic, inferior mesenteric and surrounding LNs (Fig. 1). Positron emission tomography (PET)/CT finding revealed that these LNs were hot lesions (Fig. 2). Laboratory results included an elevated serum CEA level of 16.9 ng/mL and a CA19-9 level of 109 U/mL. A biopsy of the swollen left clavicular LNs revealed moderately and poorly differentiated adenocarcinoma and immunohistochemical staining revealed caudal type homeobox transcription factor 2 (Cdx2) protein and cytokeratin 20 (CK20) protein expression (Fig. 3). The pathological findings indicated that the swollen LNs were metastatic colon cancer tumors. Subsequent colonofiberscopy uncovered depressed type (IIa+IIc) small sigmoid colon cancer with a diameter of 10 mm (Fig. 4). The pit pattern in the central portion of cancer indicated the V_I_ type according to Kudo’s classification [6] (Fig. 4). Endoscopic ultrasonography (EUS) indicated that the tumor occupied mainly the mucosal layer and had not penetrated the deep SM. Upper gastroendoscopy confirmed the absence of neoplastic lesions and cancers in the esophagus and stomach. For certainly diagnosing the primary lesion of LNs metastasis, endoscopic mucosal dissection (EMR) of the tumor was performed. Histopathological findings showed that the colon cancer was moderately and poorly differentiated adenocarcinoma that had invaded the shallow SM to a depth of 450 µm from the muscular layer of the mucosa stained by immunohistochemistry of desmin (pSM 450 µm) and revealed lymphovascular permeation of the peritumoral submucosa (Fig. 5). According to the TNM classification (International Union Against Cancer (UICC)), or the General Rules for Clinical and Pathological Studies on Cancer of the Colon, Rectum and Anus of the Japanese Society for Cancer of the Colon and Rectum [12], the colon cancer was diagnosed as stage IV (T1N2M1) or stage IV (pSM 450 µm, N3, M1), respectively. Therefore, the patient was treated with mFOLFOX6 (5-FU: 600 mg bolus injection on day 1 and a continuous 46-hour infusion of 4,000 mg from day 1 to day 2; LV: 300 mg on day 1; L-OHP: 100 mg on day 1) and bevacizumab (300 mg on day 1), every 2 weeks. After 6 courses of this regimen, CT (Fig. 6) and PET/CT (Fig. 7) findings revealed smaller metastatic LNs and decreased levels of tumor markers CEA and CA19-9. The status of the LN metastatic tumors was evaluated as a partial response (PR) according to the guidelines of the Response Evaluation Criteria in Solid Tumors (RECIST).

## Discussion

Advances in endoscopic instrumentation and techniques have increased the rates at which early colorectal carcinomas (defined as tumors with invasion limited to the mucosa or submucosa) are detected and have led to a considerable increase in the number of endoscopically removed tumors [6, 13]. Early colon cancers are defined as “invasive carcinoma that has not spread beyond the SM, regardless of the presence of blood-borne or lymphatic metastasis for the TNM classification, these lesions are represented as T1NxMx” [14]. The classification of submucosal neoplastic lesions of the colon has been developed mainly in Japan [6, 15], and SM invasion generally comprises SM1, SM2 and SM3 (invasion of the upper, middle and lower thirds of the SM, respectively) [6]. This classification has been indispensable for treating small invasive lesions of the SM, because LN metastasis is closely associated with depth of SM invasion [3, 8-10]. The reported incidence of LN metastasis in SM1 colon cancers is 0 - 5.9%, but that in SM2 and SM3 colon cancers could be as high as 11.3 - 25.0% [4, 5]. The incidence of LN metastasis is low in SM1 colon cancers, and LN metastasis-positive SM1 colon cancers are considered to be associated with regional lymphatic or venous invasion [3, 8-10], which is uncommon and can only be confirmed in resected specimens by EMR or during surgery. The incomplete endoscopic resection of SM1 cancers with regional lymphatic or venous invasion, as well as SM2 and SM3 cancers is associated with a high complication rate of LN metastasis, and often requires additional surgery including lymphadenectomy for curative treatment. The SM in surgically resected specimens can be accurately divided into three regions (SM1, SM2 and SM3), because the entire SM between the muscularis mucosae and muscularis propria is obvious. However, the SM of endoscopically resected specimens cannot always be accurately separated into three regions, because they can contain muscularis mucosae and some SM, but not muscularis propria. Therefore, the relative conventional classification for SM depth, which assumes division into three equal portions, could be confirmed only in surgically resected specimens. Thus, the relative conventional classification of SM depth is considered virtually irrelevant to endoscopically resected specimens. A novel, absolute classification of the depth of SM invasion has recently been developed in Japan to resolve these issues, and Kitajima et al. have described correlations between lymph node metastasis and the depth of SM invasion by colorectal cancers [16]. The rate of lymph node metastasis of pedunculated SM invasive colorectal cancers is 0% for head invasion and for stalk invasion when the depth of SM invasion is < 3000 µm and lymphatic invasion is negative. The rate of lymph node metastasis of non-pedunculated SM invasive colorectal cancers is also 0% when the depth of SM invasion is < 1,000 µm. These findings clarified the rates of lymph node metastasis in SM invasive colorectal cancers according to SM depth, and might also contribute to defining therapeutic strategies for such cancers. Therefore, whether the SM depth of colorectal cancers is less or more than 1000 µm is critical and SM invasive colon cancers are presently being treated according to this standard. Early colon cancers are considered to progress in two ways. The first is the relatively slow proliferation of adenoma demonstrated by polypoid growth that eventually becomes malignant. This type of cancer progression is called the adenoma–carcinoma sequence and it is considered to be the main pathway of progression. The second is the more controversial rapid and deep cancerous invasion caused by non-polypoid growth that arises *de novo* and which is not associated with the adenoma–carcinoma sequence [3]. The second type of progression was found in our patient. Non-polypoid colon neoplastic lesions are grossly classified as slightly elevated (small flat adenoma), laterally spreading (LST) and depressed [2]. Kariya et al. first described the depressed type of early colon cancers in 1977 [17]. These tend to be invasive even when small [18], and they are more malignant than flat adenomas. The incidence of SM invasion by early colon cancers is higher with depression (IIc, IIc+IIa, and IIa+IIc), than without depression or in the elevated type (Ip, Isp, and Is) [5]. One report indicates that the rates of SM invasion are 2.1%, 0.05%, 8.2%, and 29.5% for polypoid, small flat adenoma, LST, and depressed lesions, respectively [2]. Therefore, depressed lesions should always be treated carefully. Lesions confined to the mucosa or that only slightly invade the SM can be removed by EMR techniques. Additional surgical resection would be required for resected specimens with histological findings of massive SM invasion or vessel permeation to avoid a high risk of recurrence or metastasis [2]. In general, poorly differentiated adenocarcinomas are generally considered to have a high risk of LN metastasis, as well as distant metastasis [11]. It is possible that the poorly differentiated carcinoma spreads to the submucosa rapidly and more extensively [11]. One report has described distant metastasis to the liver and lung in early invasive colorectal cancer, but all of these had been associated with primary tumors in the rectum, a sessile type configuration, SM3 level of invasion, and positive regional lymphatic invasion [3]. The risk factors for metastasis included depressed type, deep SM invasion (SM2 or SM3) and regional lymphovascular permeation. Our patient had a small and depressed type (IIa+IIc) of early colon cancer. Histological findings indicated that the cancer was poorly and moderately differentiated with shallow SM invasion (pSM 450 µm) that was surmised as SM1. According to a previous report, the incidence of LN metastasis in SM1 colon cancer is extremely low. However, lymphovascular permeation of the peritumoral submucosa is one risk factor for LN metastasis. The surprising findings in our patient were the extensive metastases despite the size of the tumor, and the high level of involvement, namely the cervical, clavicular, abdominal periaortic, inferior mesenteric and surrounding LNs. According to the TNM classification or the General Rules for Clinical and Pathological Studies on Cancer of the Colon, Rectum and Anus of the Japanese Society for Cancer of the Colon and Rectum [12], our patient was diagnosed with stage IV (T1N2M1), or stage IV (pSM 450 µm, N3, M1) at initial examination. Although some reports have described early colon cancers that have invaded the shallow SM with extensive LN metastasis, they are extremely rare [19, 20]. Six courses of chemotherapy with mFOLFOX6 and bevacizumab administered as described [21], reduced these metastatic LNs and gradually decreased tumor markers of CEA and CA19-9. The status of the LN metastatic tumors was evaluated as a partial response (PR) according to RECIST.

In conclusion, we initially found suspicious metastatic LNs, the origin of which was confirmed by CT, PET and gastrointestinal endoscopy as colon cancer that had slightly invaded the shallow SM. Generally, colon cancers of up to 1,000 µm invasion rarely cause LN metastasis. We recommend that colonoscopy should be performed precisely to detect small and flat depressed lesions, since the possibility of distant metastasis even from small lesions should be considered.

## References

[1] Muto T (2000). Early colorectal cancer—concepts and clinical implications: introduction. World J Surg.

[2] Kudo S, Kashida H, Tamura T, Kogure E, Imai Y, Yamano H, Hart AR (2000). Colonoscopic diagnosis and management of nonpolypoid early colorectal cancer. World J Surg.

[3] Mainprize KS, Mortensen NJ, Warren BF (1998). Early colorectal cancer: recognition, classification and treatment. Br J Surg.

[4] Tanaka S, Haruma K, Teixeira CR, Tatsuta S, Ohtsu N, Hiraga Y, Yoshihara M (1995). Endoscopic treatment of submucosal invasive colorectal carcinoma with special reference to risk factors for lymph node metastasis. J Gastroenterol.

[5] Tsuruta O, Toyonaga A, Arima N, Sasaki E, Morimatsu M (1991). Endoscopic and radiological diagnosis of early colorectal cancer with submucosal invasion. Stomach Intestine.

[6] Kudo S (1993). Endoscopic mucosal resection of flat and depressed types of early colorectal cancer. Endoscopy.

[7] Nascimbeni R, Burgart LJ, Nivatvongs S, Larson DR (2002). Risk of lymph node metastasis in T1 carcinoma of the colon and rectum. Dis Colon Rectum.

[8] Haggitt RC, Glotzbach RE, Soffer EE, Wruble LD (1985). Prognostic factors in colorectal carcinomas arising in adenomas: implications for lesions removed by endoscopic polypectomy. Gastroenterology.

[9] Kodaira S, Yao T, Nakamura K (1994). Submucosal invasive carcinoma of the colon and rectum with metastasis: analysis of 1917 cases focused on sm invasion. Stomach Intestine.

[10] Igarashi M, Katsumata T, Kobayashi K, Sada M, Yokoyama K, Saigenji K (2000). Broadening indications for endoscopic treatment of early colorectal cancer. Dig Endosc.

[11] Nivatvongs S (2000). Surgical management of early colorectal cancer. World J Surg.

[12] Japanese Society for Cancer of the Colon and Rectum (2009). General rules for clinical and pathological studies on cancer of the colon, rectum and anus.

[13] Fujimori T, Satonaka K, Yamamura-Idei Y, Nagasako K, Maeda S (1994). Non-involvement of ras mutations in flat colorectal adenomas and carcinomas. Int J Cancer.

[14] Morson BC, Bussey HJ (1970). Predisposing causes of intestinal cancer. Curr Probl Surg.

[15] Kikuchi R, Takano M, Takagi K, Fujimoto N, Nozaki R, Fujiyoshi T, Uchida Y (1995). Management of early invasive colorectal cancer. Risk of recurrence and clinical guidelines. Dis Colon Rectum.

[16] Kitajima K, Fujimori T, Fujii S, Takeda J, Ohkura Y, Kawamata H, Kumamoto T (2004). Correlations between lymph node metastasis and depth of submucosal invasion in submucosal invasive colorectal carcinoma: a Japanese collaborative study. J Gastroenterol.

[17] Kariya J, Mizuno K, Mayama M (1977). A case of early colonic cancer type IIc associated with familial polyposis coli. Stomach Intestine.

[18] Kudo S, Tamura S, Hirota S, Sano Y, Yamano H, Serizawa M, Fukuoka T (1995). The problem of de novo colorectal carcinoma. Eur J Cancer.

[19] Lien GS, Chen CN, Cheng YS, Chen SH, Pan S, Hsieh MC, Fang CL (2001). Early colonic carcinoma with extensive lymph node metastases: case report and review of literature. Int J Colorectal Dis.

[20] Kim SA, Lee JH, Park SY, Kim H, Kim TI, Kim WH (2007). Depressed-type of early colon cancer with extensive lymph node metastasis. Yonsei Med J.

[21] Saltz LB, Clarke S, Diaz-Rubio E, Scheithauer W, Figer A, Wong R, Koski S (2008). Bevacizumab in combination with oxaliplatin-based chemotherapy as first-line therapy in metastatic colorectal cancer: a randomized phase III study. J Clin Oncol.

